# The Role of the Dietitian Within a Day Programme for Adolescent Anorexia Nervosa: A Reflexive Thematic Analysis of Child and Adolescent Eating Disorder Clinician Perspectives

**DOI:** 10.1111/jhn.70070

**Published:** 2025-05-28

**Authors:** Cliona Brennan, Georgia Green, Abigail Morgan, Julian Baudinet

**Affiliations:** ^1^ Maudsley Centre for Child and Adolescent Eating Disorders (MCCAED) The Michael Rutter Centre London UK; ^2^ London Metropolitan University London UK; ^3^ University College London Gower Street London UK; ^4^ Institute of Psychiatry, Psychology and Neuroscience (IoPPN) Kings College London London UK

**Keywords:** anorexia nervosa, day programmes, dietetics, eating disorders, nutrition

## Abstract

**Background:**

Family therapy for anorexia nervosa (FT‐AN) is the first‐line outpatient treatment for young people with anorexia nervosa (AN) in the UK. However, some require more intensive interventions, such as day programmes (DPs), which provide structured multidisciplinary care, including nutritional rehabilitation. Despite the integral role of dietitians in DPs, their specific responsibilities remain under‐researched. This study explores clinician perspectives on the role of dietitians in adolescent AN treatment to inform future research and consensus guidelines.

**Methods:**

A qualitative study using semi‐structured interviews was conducted with 11 clinicians working in one DP for young people with AN. Participants were recruited from the Intensive Treatment Programme at the Maudsley Centre for Child and Adolescent Eating Disorders. Reflexive thematic analysis identified key themes regarding dietitians' contributions to treatment.

**Results:**

Clinicians emphasised the dietitian's role in early treatment containment, reinforcing therapeutic approaches and empowering parents in meal planning and nutritional rehabilitation. Dietitians were seen as crucial in personalising treatment based on cultural and sensory needs and adapting meal plans as young people progressed. They also played a key role in guiding transitions between treatment phases, particularly from weight restoration to maintenance. However, challenges included an over‐reliance on dietitians for nutritional decisions and a ‘good cop, bad cop’ dynamic, where therapists avoided difficult conversations about food.

**Discussion:**

Findings highlight dietitians' essential role in DP treatment for AN but suggest that excessive reliance may limit therapist autonomy. Strengthening collaboration through shared decision‐making and bidirectional learning is recommended. Further research should explore these dynamics across diverse settings.

## Summary

- This study explores clinician perspectives on the role of dietitians in adolescent anorexia nervosa (AN) treatment to inform future research and consensus guidelines.
- Dietitians were recognised as having an essential role in day patient treatment for AN; however, challenges identified included a potential over‐reliance on dietitians for nutritional decisions and a ‘good cop, bad cop’ dynamic, where therapists avoided difficult conversations about food.
- Strengthening collaboration through shared decision‐making and bidirectional learning is recommended

## 1 Introduction

Family therapy for anorexia nervosa (FT‐AN) is the first‐line recommended outpatient treatment approach for young people (< 18 years) diagnosed with anorexia nervosa (AN) in the United Kingdom (UK) [1]. Although the positive effects of outpatient FT‐AN are broadly cited in the literature, more intensive treatment options can often be needed to support remission. A range of more intensive eating disorder (ED) treatments exist, including inpatient care [2], residential treatment [3], day programmes (DPs) and Partial Hospitalisation Programmes (PHPs) [4], intensive multi‐family therapy [5, 6] and intensive outpatient programmes (IoPs) [7].

Inpatient units provide specialist ED care and are well placed to support medical stabilisation and weight restoration, both essential components of care in preventing morbidity and mortality [8]. Further benefits of prolonged inpatient hospitalisation, post‐medical stabilisation, are disputed in the literature [9, 10]. For those who are medically stable, DPs have equivalent outcomes to inpatient treatment [11] and may have better outcomes at follow‐up [12]. As such, they are becoming increasingly widespread internationally. Although DPs vary in their content and structure internationally, components that are broadly similar across programmes include nutritional rehabilitation, meal support, intensified therapeutic input (when compared with typical outpatient treatment), parent and carer involvement and a group‐based (rather than individual) approach [4, 12]. DPs typically operate 4–7 days/week and have an average length of stay between 3 and 18 weeks [13], throughout which period the young person (YP) remains at home and is predominantly supported by their parents to recover. DPs facilitate increased family involvement (a cornerstone of FT‐AN) in treatment and are cost‐effective when compared with inpatient admission for young people with AN [14].

The use of a multidisciplinary team (MDT) working is advised across all treatment settings in AN (NICE, 2017) and is crucial in higher levels of care, such as inpatient and DP settings, where physical risk is generally greatest. Medical and nutritional management (alongside therapeutic interventions) of relevant risks are central parts of AN treatment, delivered by professionals qualified in psychiatry, medicine and nutrition [15]. Although dietitians are well placed to support nutritional‐related issues arising from AN and are considered a central part of the MDT in DPs, their specific roles and responsibilities remain under‐researched and undefined.

In the treatment of adolescent AN, dietitians are considered to play an integral role in extending the nutritional knowledge base of the MDT, providing nutritional psychoeducation in collaboration with therapists and in devising meal plans and guidance which are used in the initial stages of treatment [16]. Benefits of dietetic input have been reported by both clinicians and families [17, 18] and may support those who do not respond early in treatment [19]. Aspects of dietetic care, in the inpatient and outpatient setting, that are perceived as most helpful include person‐centeredness, collaboration, trust and specialist expertise [18, 20].

There is little clarity on the role and contributions of dietitians in DP settings for YP with AN [21]. The limited available evidence suggests that dietitians, similar to inpatient and outpatient settings, are key members of the treating MDT [22]. Dietitians are central to nutritional rehabilitation and nutritional counselling during DP treatment, both of which have been identified as core elements of the multimodal approach required to effectively restore physical health in YP with AN [21]. Individualised meal plans, created collaboratively with the family, food portioning guidance and parent coaching are advocated as important nutritional interventions in DPs, complementary to MDT treatment of the illness. Practical food groups, facilitated by dietitians and nursing staff, have also been perceived as helpful by DP attendees, although research on these interventions remains limited [23].

Further evidence is needed to guide nutritional interventions and their effectiveness, across settings and particularly in DPs where evidence is severely limited [24]. Currently, a sparse evidence base exists to validate the efficacy of nutritional interventions in this setting. No empirical studies have been conducted, to date, which assess the effect of nutrition counselling on treatment outcomes in DP for YP with AN. In the absence of such studies, the ability to advance our understanding of the role of dietitians in DP treatment remains limited. This study aimed to gather perspectives from experienced multidisciplinary clinicians, working within one DP for YP with AN, on the role of the dietitian in this setting. The objective of the study was to take an initial step towards gathering evidence that supports further research in this area and will aid the development of consensus guidelines on this role.

## 2 Methods

### 2.1 Ethical Considerations

Approval for this project was obtained from South London and Maudsley service evaluation and audit committee (approval number 330). All participants provided written consent.

### 2.2 Study Design

Professional's perception and experience of the role of the dietitian in the Intensive Treatment Programme (ITP) were explored using semi‐structured interviews. Various methods for sample size calculation in qualitative research exist [25]. Data saturation is one method that has been proposed to calculate sample size [26]. However, this method has been disputed by some authors [27]. Alternative proposed guidelines for reflexive thematic analysis, proposed by Braun and Clarke [28] categorise sample size suggestions by the type of data collection and the size of the project. For small projects, 6–10 participants are recommended for individual interviews [28]. Sample size in this study was informed by this guidance.

Clinicians working across one site, ITP, were invited to take part in the study by members of the research team. Those interested in taking part were recruited to the study and assigned a study ID. Individual interviews were intentionally used, rather than focus groups, as this was thought to encourage openness and reduce the possibility of demand characteristics.

### 2.3 Treatment Setting

ITP is a large, publicly funded, specialist child and adolescent eating disorder service. The service is a DP for up to 12 YP aged 11–18, with predominantly restrictive eating disorders. The primary aim of ITP is to support young people and their families in making progress toward recovery by teaching valuable skills that can be applied to daily life while remaining at home. The programme runs 5 days per week (9:45 AM to 4:00 PM), including one extended day for a dinner with the families. The intensity of attendance is tailored to individual clinical needs, with young people often starting on a full‐time basis. Another key focus is on reintegration into school, gradually reducing days at ITP, and transitioning to outpatient care to continue recovery. See recent articles for a description of ITP structure and outcomes and families experience of the programme [29, 30, 31, 32]. The programme is designed to finish within two ‘rounds’ (each round is equivalent to a UK school term), providing a structured timeframe for treatment over approximately 12–14 weeks. The MDT at ITP is comprised of psychiatrists, psychologists, assistant psychologists, nurses, family therapists, art therapists and a dietitian.

In ITP, a 60‐min dietetic assessment takes place with each new admission to the programme. Both parents, the YP, the treating therapist and the dietitian attend this first dietetic session which includes a comprehensive assessment of dietary intake and setting of initial nutritional goals (i.e. for most young people, this will be in the form of a meal plan). All YPs are reviewed weekly in an MDT meeting, in addition to dietetic reviews which take place as often as clinically indicated (i.e. troubleshooting issues with meal plan, weight gain and when moving to later stages of treatment such as independent or intuitive eating). A weekly ‘food group’ takes place each Monday in ITP, focusing on nutritional psychoeducation and practical food preparation sessions. Young people attend this group weekly with the support from a dietitian, a nurse therapist and an assistant psychologist.

### 2.4 Sample and Recruitment

All clinicians (*N* = 16) working in ITP who met the inclusion criteria during the study recruitment period (February to June 2024), were invited to participate. Inclusion criteria were National Health Service (NHS) clinician, experience working with restrictive eating disorders (> 4 months) and experience of working with a dietitian. Exclusion criteria were less than 4 months of experience working with restrictive eating disorders and no experience working with a dietitian. Written informed consent was obtained from all participants.

### 2.5 Procedure

Interviews were conducted via the Microsoft Teams platform [33]. All interviews were audio‐recorded, from which written verbatim transcripts were created. All identifiable information was removed during the transcription process. A semi‐structured interview format was used, with each participant being asked the same set of open‐ended questions (see Appendix for interview schedule). Each interview lasted a maximum of 45 min and were scheduled at a time that was convenient for each participant. Interview questions were created in consultation with the supervisory team.

The topic guide was designed by authors C.B., J.B. and G.G. and used open‐ended questions to explore participants' views and experiences of the role of dietetics in DPs. Data were collected between January and June 2024.

### 2.6 Analysis Plan

Qualitative data were analysed using reflexive thematic analysis for identifying, analysing and interpreting data‐driven themes, following the six phases outlined by Braun and Clarke. Analysis was carried out within a critical realist framework, which views meaning and experience as subjective and influenced by social and cultural context. Transcripts were read and reread to ensure data familiarisation. Transcripts were then coded by three researchers (C.B., G.G. and A.M.) to help interrogate the data. Through collaborative discussions, initial codes were sorted into preliminary themes. Themes were developed through reflexive engagement with the data. Themes were cross‐checked with participants, and comments or feedback were used to adapt the themes to accurately reflect the views of participants.

### 2.7 Reflexivity Statements of Analysing Authors

G.G. is a cisgender white female studying a masters in eating disorders and clinical nutrition at University College London. Having studied the treatment of FT‐AN and completed placements days observing at the MCCAED, she understands and is aware of the role taken by the dietitian in this team. It is understood that the conclusions drawn could be influenced by these experiences, resulting in research bias. A reflexive approach has been taken in aim of preventing against this.

A.M. is a cisgender white female working as an assistant psychologist delivering FT‐AN at the MCCAED. She has experience working as a member of the MDT within this service, collaborating with all clinicians, including the dietitian. She understands her role and experiences within the team used for the research sample could bring bias to the data analysis. A reflexive approach has been taken in aim of preventing against this.

C.B. is a cis‐white female working as a dietitian within community services for child and adolescent ED, she understands and is aware of the roles and responsibilities that surround being a dietitian in this service. The questions asked within the interviews and the themes drawn from responses will inherently contain biases due to their role in their team. The results and conclusions come from their perspective and their awareness of these biases have been considered.

## 3 Results

### 3.1 Sample

A total of sixteen eligible participants were approached (two psychiatrists, four psychologists, three nurses, one family therapist, four assistant psychologists and two psychological practitioner trainees). Eleven consented to participate and completed interviews. The included sample consisted of one psychiatrist, three nurses, three psychologists, two assistant psychologists and two psychological practitioner trainees). All participants had > 4 months of experience working within eating disorders in the NHS.

### 3.2 Qualitative Findings

1. Affirmation of treatment approach
  Dietetics had a key role in affirming the overall treatment approach, in particular in the initial stages of DP treatment. Clinicians felt the dietitian scaffolded the engagement of the family with the therapist and aided the creation of a stable base in the DP intervention. Containment was supported by including dietetic input early in treatment. Clinicians agreed that the specialist and individualised dietary guidance that was offered by the dietitian supported parental confidence in feeding their child and trust in the treating MDT.
  1a. Supporting a stable base
  A core theme evident amongst all participants was the importance of dietetic input in the early stages of DP treatment. Dietetic input was considered helpful in containing anxiety during the initial period of treatment, in scaffolding engagement between the team and family. Direct dietetic involvement was viewed as a tool to affirm the treatment approach employed during the early stages of DP treatment, typically focused on weight gain. During these initial weeks, parental empowerment to refeed their child is crucial. The collaborative nature of creating a meal plan together with the dietitian, therapist, parents and YP within the dietetic session was perceived to unite individuals together in managing eating disorder symptoms. This supported the creation of a stable base required to effectively treat the illness.
  At assessment (dietetic input is important) in terms of making sure that we can support the family appropriately at the start of treatment in getting them on board, building engagement, psychoeducation challenging eating disorders.
  [2]
  I think the expertise that is brought by a dietician [in the early stages] is critical then because…because you need to meet a very strong illness, like a very strong argument for change. And I think the dietitian can bring that argument. And prompt change, so I would probably say maybe the beginning (dietetics is important).
  [6]
  1b. Containment
  Direct dietetic support was perceived by clinicians to be integral in the containment of the system. Empowering parents in their ability to support their child and provide adequate and balanced nutrition was seen as one benefit of dietetic involvement during DP treatment. The expertise of the dietitian in relation to food and nutrition was thought to help parents feel more confident in their own skills and knowledge. Input from the dietitian generally promoted and further strengthened messages from the treating therapist, reinforcing the narrative around early re‐feeding and the importance of challenging eating disordered behaviours.
  (The dietitian) is quite empowering…. reiterating to parents that they already know what they're doing a lot of the time and that maybe they need some tweaks and some changes, but also putting trust into parents because they feed themselves, they feed their children.
  [1]
  It's not just me as the main clinician, it's like me and a highly trained specialist in that area being able to kind of challenge the anorexia.
  [2]
2. Systemic meaning of individualisation
  This theme further highlighted the valuable contribution of dietetics in DP treatment. Clinicians reflected on the impact of tailored dietary guidance early on engagement, trust and progress in DP treatment. Participants agreed that the dietetic input could often be a vehicle for change when progress had halted and punctuated different stages of treatment in this setting. Given the diverse needs of the population served, and the dynamic needs of each family through these stages of treatment, the ability to individualise dietary guidance was viewed as essential.
  2a. Patient‐centred care to facilitate engagement
  Participants considered dietetic input to support the individualisation of treatment plans and ensure that the diverse needs of each YP were held in mind. Individual needs of each family and YP were assessed and considered by the dietitian in the context of their other needs and MDT work, enabling a YP‐centred plan to be created and implemented by the treating therapist and family. Engagement was further facilitated through this approach, allowing the specific needs of each family to be met through collaborative decision‐making between the family and professionals.
  In the kind of co working that's been done, that's been invaluable at assessment. Is like the dietitian thinking about allergies, thinking about sensory needs, thinking about anything else that may impact on being able to follow a meal plan.
  [2]
  I think young people appreciate the collaborative approach that our dietitian takes with creating those meal plans… I think definitely with it being the whole family are involved in those decisions that helps with getting them engaged rather than just giving them like a blanket meal plan. That's for everyone. I think it's really helpful to take more of a… patient focused approach.
  [9]
  2b. Meeting diverse needs
  Participants said that direct involvement for the dietitian was particularly important in facilitating a culturally sensitive approach. Meal planning, psychoeducation and problem solving based on the diverse needs of each family was aided by the dietitian. The varied cultural needs of each family were discussed with the dietitian during discussions around food and setting expectations around eating and mealtimes. This benefited engagement with the treatment plan.
  Coming up with a meal plan that fits for that young person and their family, how does that incorporate their cultural differences and things like that, that are super important. We can't just expect everybody to follow a Western meal plan if actually culturally that's just very different to what they would normally do.
  [3]
  I think with it being the whole family involved in those decisions that helps with getting them engaged rather than just giving them like a blanket meal plan. That's for everyone. I think it's really helpful to take more of a… patient focused approach.
  [9]
  2c. Catalyst for change
  Participants said that another key role of the dietitian in DP treatment was during the transition from weight restoration to meeting broader needs and eating disorder symptomatology. Clinicians felt that bringing the dietitian into sessions punctuated the change in treatment focus. This was noted when moving from weight restoration in the initial phases of treatment onwards to later stages of treatment when weight restoration was less of, or no longer, a priority. Additionally, clinicians reported that dietetic sessions were helpful during periods where progress had stagnated, and the family was no longer moving forward. Dietetic involvement at these times was perceived to facilitate direct communication about current barriers to progress and specifically address the need to make changes and move forward in treatment.
  You might not need to see them (the dietitian) then for a while, but then it's thinking about when you're in that next phase really wanting to pass that responsibility back [to the young person]. I think again it becomes so important for the dietitian to be the person that's kind of like leading that or guiding that process.
  [3]
  The dietitian is important in then reducing the meal plan that they're on and thinking of a meal plan i.e. no longer maybe weight restoration, but more just like maintaining and thinking through changes. Thinking through those changes with the young people and maybe even more independent eating or intuitive eating kind of thing would look like for someone. And again, the dietitian would be the one with the knowledge of how your body might then react to these things like would you be displaying more hunger cues? So again, the dietitian would be really that stage treatment.
  [6]
  I think along with kind of the first initial meeting, it's maybe when the young person is maybe not making as much progress weight wise, or there seems to be like a bit of stuckness and I think helping to review how things are going, I think i.e. valuable to kind of sit down with the family and have a review of how things are going and how they can kind of help support to make change to get out of this stuckness that maybe they might be experiencing. I think i.e. where I found a dietician really valuable.
  [9]
3. Interprofessional Collaborative Practice
  The theme of interprofessional collaborative practice underscores the importance of collaboration between therapists and dietitians in the treatment of AN, ensuring all disciplines contribute meaningfully to patient care. This theme stresses the crucial nature of collaborative practice across disciplines in the treating MDT. In the DP setting, given the multiple daily interactions of various clinicians with each family team working and clear, consistent communication is necessary to provide effective and streamlined care. Additionally, joined‐up working reduces the risk of team splitting. Mutual and bidirectional learning between the dietitian and other disciplines was seen as an important element, alongside a joined‐up and supportive team approach.
  3a. Promoting mutual learning
  This subtheme highlights the importance of promoting mutual learning across disciplines. Over‐reliance on dietitian expertise may reduce therapists' and family members' autonomy, leading them to disengage from areas they perceive as the ‘dietitian's domain’. Several clinicians expressed trust in the dietitian and confidence in their decisions, often accepting them without questioning the rationale behind them. While dietitians play a critical role in providing nutritional expertise, promoting shared responsibility and mutual learning is essential for maintaining a cohesive and effective MDT, and supporting independence from services and discharge.
  I think that's so valuable because I know that I can just rely on and trust whatever she has to say about the topic.
  [1]
  My skills are in kind of like therapy. I don't necessarily know so much about kind of like nutrition and dietetics and kind of what calories things are and kind of health‐wise, what's needed for somebody with anorexia… it just kind of means that I don't necessarily have to worry about it so much.
  [3]
  We would be kind of lost without them being able to guide us.
  [6]
  I think we do rely on the dietitian's expertise, but I also think there's a sense of that responsibility is held by the dietician, so we almost wouldn't want to get involved because we know we don't have the expertise that the dietician has.
  [8]
  3b. ‘Good cop, bad cop’ dynamic
  Another consensus that emerged was a *‘*good cop, bad cop dynamic*’* in the collaboration with the dietitian, with the dietitian often adopting the *‘*bad cop’ role. One participant explicitly referenced this dynamic, while several others described a similar pattern in which the responsibility for making and communicating challenging nutritional decisions is placed solely on the dietitian, rather than being viewed as a shared decision within the MDT. Therapists noted often deferring to the dietitian's authority when faced with difficult conversations about nutrition or meal plans, using phrases that framed the dietitian as the clinician making the decisions. This dynamic helps reduce therapists' discomfort and allowed them to avoid taking responsibility in leading distressing conversations with young people and their family members. While useful at times, it was also described as creating the potential for splitting within the team. As one participant noted, this could make therapists distance themselves from the more challenging aspects of care in AN.
  I think sometimes she can be viewed a little bit as like bad cop that she comes in and talks about the food, and then we get to talk about, like, other stuff, which is maybe more kind of like therapeutic…. I think there is the chance, sometimes, for there to be that kind of like splitting in the team of, like, liking the mini team, the therapist, but not liking the dietitian because they come in with those firm boundaries.
  [3]
  With anorexia, the kids question you so much about what they're having and whether it's the right thing to have. And it's nice to be like, oh, this isn't on me. Like the dietitian has told me, this is what you need.
  [4]
  I think I've referenced it, but I think just having the phrase of, oh, yeah, you can definitely bring that up with the dietician in your next session with them. I've used that a lot of times. It basically serves to remove me from a decision when I'm unsure of the decision.
  [6]

## 4 Discussion

The aim of the current study was to advance our understanding of the role of the dietitian in DP treatment through gathering perspectives from experienced clinicians working in this setting. Overall, dietetic input was considered a valuable resource within the MDT. Collaboration was a common thread through all themes that were generated from the clinicians interviewed. Through working together with clinicians and families, the dietitian aided containment of the system and the delivery of a patient‐centred approach. Clinicians viewed dietetic input as an essential ingredient in effectively combating AN and facilitating progression through treatment.

Access to dietetic expertise was most valued during the initial phases of DP treatment, where nutritional rehabilitation and weight restoration are most often prioritised [30]. Nutritional guidance, delivered by an expert in nutrition, facilitated engagement with the MDT by promoting the therapeutic approach with scientific rationale and individualised meal planning. Clinicians felt that this aided the containment of anxiety across the system. This fits with previous qualitative data on the young person and parent experience of DP treatment, which suggests communication and connection with other young people, parents and staff in the programme are key to promoting change [32, 34]. Family‐led and collaborative interventions have previously been reported as valuable in dietetic treatment [35]. Building the initial meal plan together was described as a collaborative and unifying activity in the current study, as well as in the literature [24, 36].

Dietitians have previously been identified as highly valued professionals by families in the treatment of AN [17]. Dietitians were considered to have a core role in empowering parents and increasing parental confidence in feeding their unwell YP. Specific contributions of the dietitian during DP treatment, identified in this study, centred around the individualisation of treatment. The ability to tailor meal plans, adapt nutritional guidance to YPs needs and contextualise nutritional recommendations as YP moved through phases of treatment was highly regarded. The role of the dietitian in delivering individualised and patient‐centred care effectively has been widely reported in literature on this topic to date [35, 37].

Interprofessional collaborative practice care during DP treatment is highly beneficial, as the overlap of roles among healthcare providers enhances the consistency of treatment and reinforces key recommendations [38]. Clinicians emphasise the importance of all team members contributing their expertise within a cohesive, joined‐up team. This mirrors the importance of individualised formulation in outpatient FT‐AN [39]. In the DP setting, the intensity and frequency of patient contact inherently create opportunities for role overlap among MDT members. For instance, the dietitian, therapists and nursing staff all provide meal support and co‐facilitate therapeutic groups. This can lead to a splitting dynamic in DP, where therapists disengage from aspects of care they perceive as outside their role and over rely on the dietitian's expertise. However, some clinicians viewed this collaboration as beneficial and can be managed with consistent messages and a unified approach to treatment [36].

The results also suggest that dietitians can often be placed in a ‘bad cop’ role when therapists do not actively participate in nutritional discussions. To foster a more cohesive team dynamic and maintain therapist autonomy, shared decision‐making and bidirectional learning between dietitians and therapists are essential, ensuring challenging conversations are a collective effort rather than the sole responsibility of the dietitian. The collaborative nature of the DP model is viewed as integral to implementing the FT‐AN approach, with all team members working together to address the complex interplay of factors maintaining the eating disorder. While the dietitian's expertise in nutrition is a crucial and distinct component of their role, their ability to input within the multidisciplinary framework is a key strength of the DP model. This differs from outpatient settings, where dietetic involvement is considered on a case‐by‐case basis [18].

### 4.1 Strengths and Limitations

This is the only study to qualitatively explore clinicians' perspectives on the role of a dietitian within a family‐focused DP treatment setting. A strength of the study is that participants represented various professions within the MDT, expanding on previous studies that have focused solely on the dietitian's perspective of their role in outpatient FT‐AN.

One limitation of this study is that the findings were based on a convenience sample of clinicians who self‐selected to participate. This could introduce self‐selection bias, as individuals who volunteered may have had more positive or negative views on the role of dietitians in FT‐AN, which may have influenced their decision to participate. Additionally, the study was conducted at a single centre, which means the findings may not be representative of other similar DP offering a family‐informed treatment approach. As such, the results are context‐specific and may not capture the experiences and perceptions of dietitians in services other than MCCAED‐ITP. This study also did not capture the perspective of the dietitian on their own role. More data are needed to understand whether these experiences are common across centres and internationally.

## 5 Conclusion

To avoid over‐reliance on dietitians and ensure a more cohesive team dynamic, it is important to foster bidirectional learning between dietitians and therapists. Both parties should engage in shared decision‐making and mutual learning to maintain therapist autonomy and avoid the ‘good cop/bad cop’ dynamic. While dietitians will continue to provide expertise, therapists should actively engage in and support nutritional discussions with the YP, ensuring that challenging conversations are seen as a team effort rather than the sole responsibility of the dietitian.

## Clinical Recommendations

1. **Integrate Dietetic Input Early in DP Treatment to Establish a Stable Foundation:** Involving dietitians early in the DP treatment process can support the initial stages of care, particularly during the critical phase of re‐feeding. Their involvement in co‐developing meal plans with the family, therapist and YP can help reduce anxiety, build engagement and ensure a unified approach to treatment. This collaboration fosters engagement with the whole MDT and helps to create a stable foundation i.e. essential for effectively addressing AN.
2. **Promote Patient‐Centred, Individualised Care:** Dietitians play a crucial role in personalising treatment plans to address the diverse needs of each YP and their family. By considering cultural differences, sensory needs and specific dietary preferences, dietitians have a role in ensuring the treatment plan is relevant and tailored to the YPs circumstances. This individualisation enhances family engagement and ensures the treatment approach is both practical and culturally sensitive.
3. **Support Transitions Between Treatment Phases and Facilitate Change:** The dietitian's role can support transitions between treatment phases, particularly when shifting from weight restoration to maintenance or independent eating. In these instances, dietitians help guide families through adjustments to meal plans, promote the development of intuitive eating and address challenges that may arise when progress stagnates. Their expertise in nutrition allows them to provide critical support in moving forward with treatment, ensuring the plan evolves as the YP progresses.
4. **Foster Collaborative, Shared Responsibility While Avoiding Role Splitting:** While dietitians bring essential expertise to nutritional care, it is important to maintain a collaborative approach within the MDT. Over‐reliance on the dietitian's knowledge and skill may reduce therapists' confidence and autonomy. This can contribute to a ‘good cop, bad cop’ dynamic, where therapists avoid challenging nutritional decisions. Encouraging mutual learning and shared responsibility ensures that all team members remain actively engaged in all aspects of care, supports families not to become overly dependent on professionals, preventing division and ensuring a cohesive treatment approach.

## Author Contributions

Cliona Brennan and Julian Baudinet were responsible for the conception and design of the study. Georgia Green was responsible for recruiting participants and collecting data. Cliona Brennan, Georgia Green and Abigail Morgan were responsible for the analysis of data and preparation of draft version of the manuscript. All authors contributed to the final version of the manuscript and associated results and documents.

## Ethics Statement

Approval for this project was obtained from South London and Maudsley Child and Adolescent Mental Health Services (CAMHS) service evaluation and audit committee (approval number 330).

## Consent

All participants provided written consent.

## Conflicts of Interest

The authors declare no conflicts of interest.

### 1 Peer Review

The peer review history for this article is available at https://www.webofscience.com/api/gateway/wos/peer-review/10.1111/jhn.70070.

## Supporting information

Supplementary Information

## Data Availability

The data that support the findings of this study are available on request from the corresponding author. The data are not publicly available due to privacy or ethical restrictions.
