# Supplementary figures and images for: The Role of the Dietitian Within a Day Programme for Adolescent Anorexia Nervosa: A Reflexive Thematic Analysis of Child and Adolescent Eating Disorder Clinician Perspectives
Source: J Hum Nutr Diet. 2025 May 28;38(3):e70070. doi: 10.1111/jhn.70070 (PMC12120378; doi:10.1111/jhn.70070)

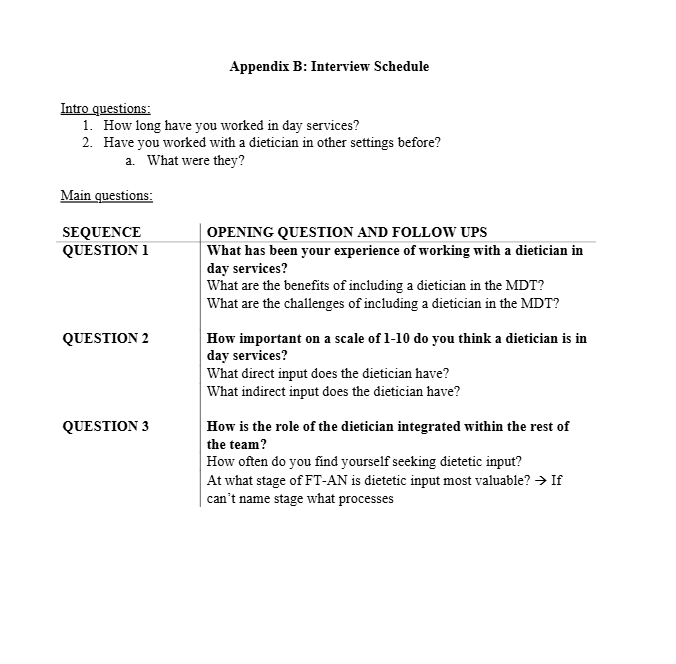

Supplement: Supplementary file 1 — Supplementary Information [file JHN-38-0-s001.docx]
